# Therapeutic and Prophylactic Effect of the Experimental Bacteriophage Treatment to Control Diarrhea Caused by *E. coli* in Newborn Calves

**DOI:** 10.1021/acsinfecdis.1c00010

**Published:** 2021-04-05

**Authors:** Mohammed Mijbas M. Alomari, Marta Dec, Anna Nowaczek, Andrzej Puchalski, Andrzej Wernicki, Cezary Kowalski, Renata Urban-Chmiel

**Affiliations:** † Sub-Department of Veterinary Prevention and Avian Diseases, Institute of Biological Basis of Animal Diseases, University of Life Sciences, Akademicka 12, 20-033 Lublin, Poland; ‡ University of Al Muthanna, Faculty of Veterinary Medicine, Al Sumawy City Main Street, Al Muthanna 66001, Iraq; § Sub-Department of Pharmacology, Toxicology and Environmental Protection, Department of Preclinical Veterinary Sciences, University of Life Sciences, Akademicka 12, 20-033 Lublin, Poland

**Keywords:** bacteriophages, bacteria, calves, diarrhea, therapy

## Abstract

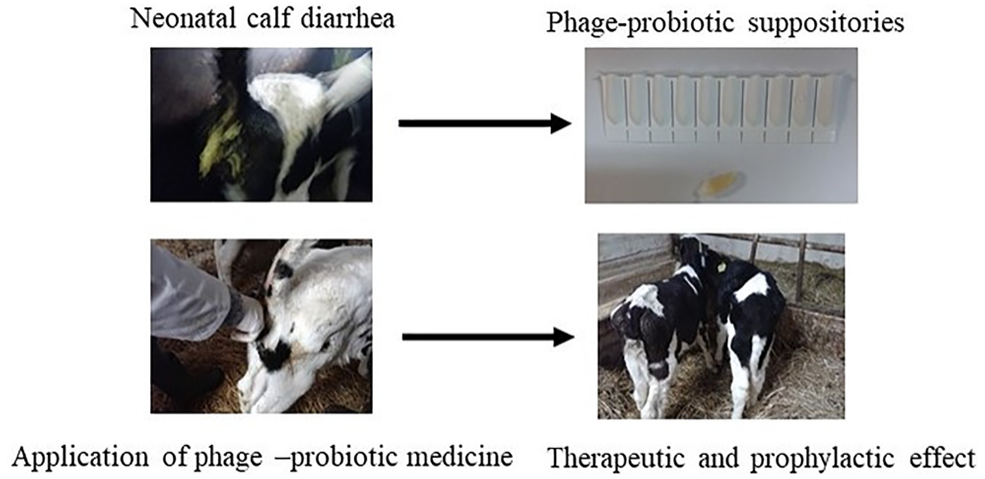

The prevalence of antibiotic-resistant bacteria causing neonatal diarrhea in calves has become a serious problem in the control of infection. Due to increasing antibiotic resistance, bacteriophages with probiotics are considered the best alternative. The aim of the study was to evaluate the use of a suppository containing probiotic strains of *Lactobacillus* spp. and bacteriophages specific for pathogenic *E. coli* in young calves with diarrhea. The study evaluated therapeutic and prophylactic effects (specific and nonspecific humoral response). The study was carried out on 24 female HF calves, aged 2 to 7 days and weighing from 35 to 46 kg. The calves were divided into four groups (*n* = 6) as follows: Group 1, healthy control that received no medicine; Group 2, positive control with diarrhea; Group 3, healthy calves that received medicine; Group 4, calves with diarrhea that received medicine. The animals received suppositories containing *Lactobacillus* spp. and bacteriophages specific for pathogenic *E. coli* for 5 days. On the first day, the calves received the suppositories twice—in the morning and 12 h later; subsequently they were administered once a day. The health status of the calves was observed for 11 days after the first application of suppositories. A protective and preventive effect of the experimental therapy was obtained in the research. The probiotic-phage suppositories reduced the duration of diarrhea in calves, completely eliminating it within 24–48 h after use. The therapy stimulated the activation of immune mechanisms in calves, which translated into an enhanced specific and nonspecific response and increased resistance to infection.

Neonatal calf diarrhea, caused by various infectious agents, including viruses (rotaviruses and coronaviruses), parasites such as *Cryptosporidium* spp., and bacteria, including *E. coli* K99, is one of the most important diseases in newborn calves during the first few weeks of life.^1,2^ It has been documented^3,4^ that pathogenic *E. coli* causes diarrhea in calves during the first week of life, while it is viruses and parasites that primarily affect older calves. Diarrhea in calves has a major impact on the economic viability of cattle herds worldwide. In France, the mortality of dairy heifers between 3 days and one month of age is estimated at 5.7%,^5,6^ while in the USA it is more than 6.9%.^7^ The most common pathotypes of *E. coli* strains associated with neonatal calf diarrhea are enterotoxigenic (ETEC) and enteropathogenic (EPEC) *E. coli*, which studies suggest are responsible for high morbidity and mortality rates.^8−10^

Major difficulties in treating diarrhea caused by pathogenic strains of *E. coli* in neonatal calves are associated with the duration of therapy and the use of the right antibiotic. *E. coli* is an important causative agent which has shown antimicrobial resistance.^2^ Commonly used antibiotic treatment can significantly contribute to immunosuppression in calves, increasing their susceptibility to infections. It may also increase bacterial resistance, making effective elimination of infections more difficult.^11,12^ The most commonly used antibiotics include β-lactams, aminoglycosides, fluoroquinolones, and tetracyclines.^13^ These antibiotics are also used in humans, which is a serious problem for public health and necessitates the search for alternative therapies to antibiotics. Moreover, legislative restrictions on the use of antibiotics, including β-lactam antimicrobials such as penicillins and cephalosporins, polymyxins, fluoroquinolones, and aminoglycosides, create the need for auxiliary measures to control infections.^14^ Therefore, in addition to traditional antibiotic therapy, many studies have discussed alternative methods of treatment using natural substances such as garlic, aloe vera or other plant extracts, lactoferrin, or probiotics.^15,16^ Antibiotic resistance in bacteria has emerged following the widespread of use of antibiotics to treat numerous infections in humans and animals.^14^ Alternative methods are sought for the elimination of pathogens, and treatment of diarrheic calves with bacteriophages in combination with probiotics having antimicrobial potential is regarded as the best alternative to antibiotics. This is due to the significant action of bacteriophages destroying the integrity of the bacterial biofilm through destruction of the cells producing the biofilm matrix, which has been confirmed in numerous experiments.^17−19^

In view of the above, the aim of the study was to evaluate the therapeutic and preventive effect of suppositories containing *Lactobacillus* spp. and bacteriophages specific for pathogenic *E. coli* in young calves with diarrhea.

## Results and Discussion

**Figure 1:**
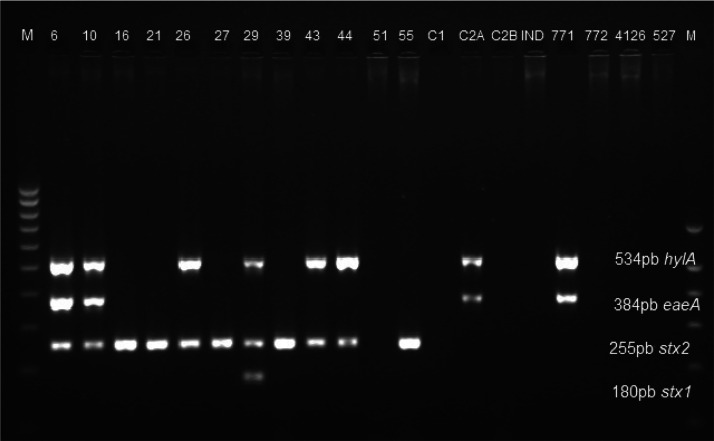
Detection of virulence genes in *E. coli* isolates used for in vitro and in vivo testing by multiplex PCR. Legend: M, molecular weight marker (100–1000 bp); *E. coli* strain numbers are given in individual lines.

The results of the genetic analysis of the presence of virulence genes in the *E. coli* strains are presented in Figure 1. The results of multiplex PCR confirmed the presence of virulence-associated genes (*stx1*, s*tx2*, *eaeA*, and *hlyA*) in 13 of 20 *E. coli* strains (65%) used for the in vitro tests. Eleven *E. coli* strains were positive for *stx* genes encoding Shiga toxin—one strain had *stx1*, and 11 had *stx2*. One *E. coli* strain (no. 29) contained both the *stx1* and *stx2* genes. Four strains (20%) contained the gene *eae* encoding intimin; the *hlyA* gene encoding hemolysin was present in 8 isolates (40%); and 4 strains (20%) contained *saa*, corresponding to an outer membrane protein that plays a role in autoagglutinating adhesion.

Ten phages were obtained, but only three showed strong lytic properties against all pathogenic *E. coli* strains. All qualified phages belonged to the family *Myoviridae* and were characterized by lytic titer stability in a pH range from 3.5 to 6.0 and a broad spectrum of antibacterial activity against Stx and K99 *E. coli* strains owned by our unit. Only three bacteriophages (φ26, 27, and 29) causing complete lysis of bacteria in the form of plaques on two-layer plates were used for further study.

**Figure 2:**
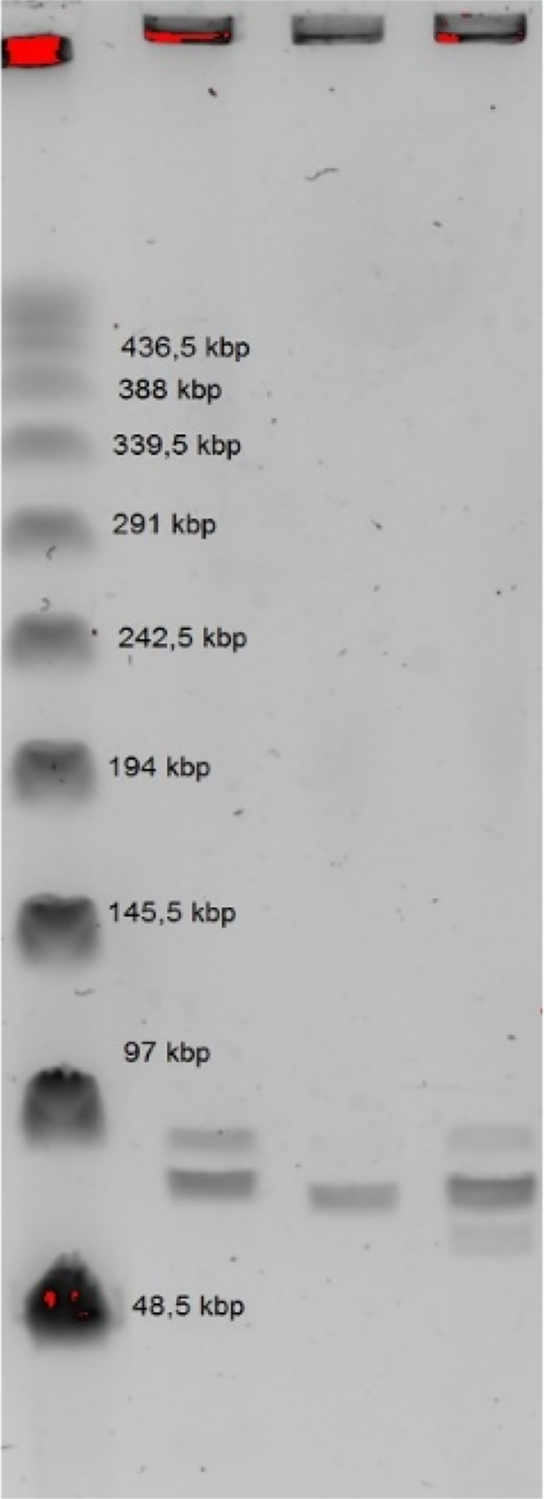
Pulsed-field electrophoresis (PFGE) of undigested phage DNA. Legend: The lanes contained: 1, Marker II (485–48.4 kb), 2, φ26; 3, φ27; 4, φ29.

The genome size of undigested phage DNA in pulsed-field electrophoresis (PFGE) was estimated at 93 ± 3 kbp (Figure 2).

A detailed characterization of the phages is presented in Table 1.

**Table 1:**
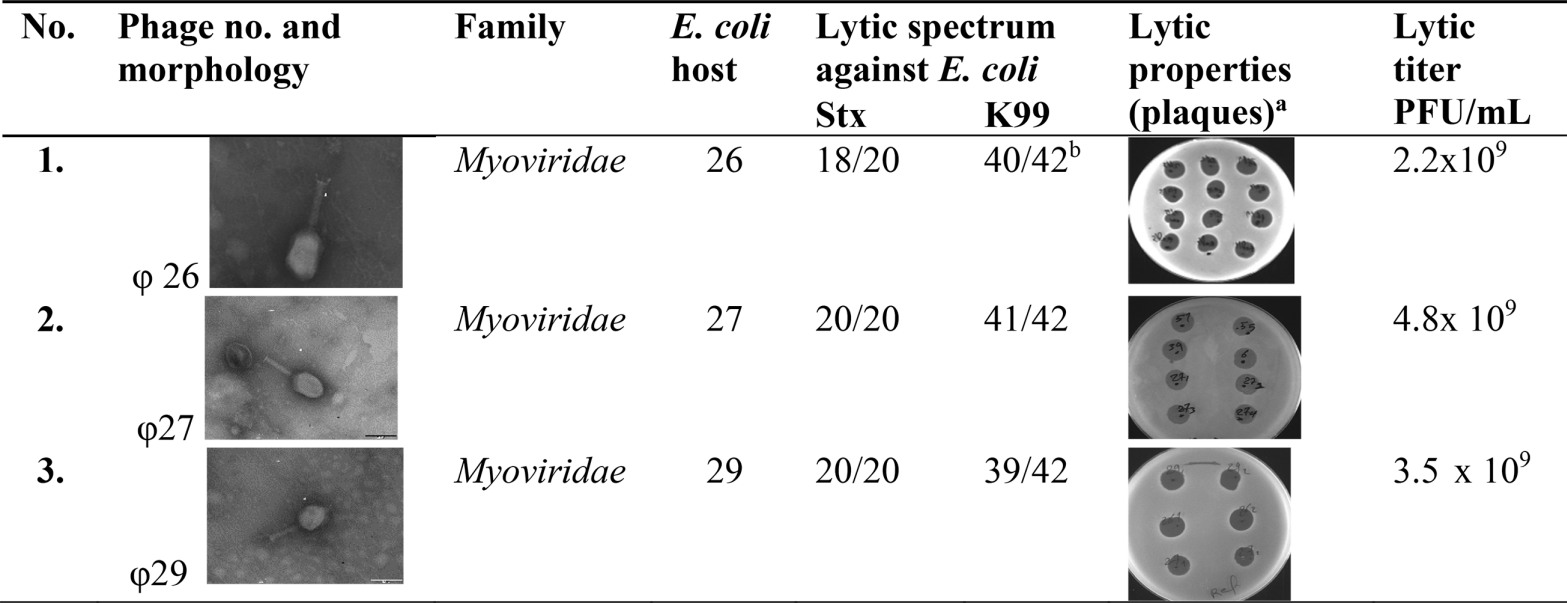
Morphology and Lytic Titers of Bacteriophages Specific for Stx *E. coli* and K99 *E. coli* Strains Isolated from Cattle

a Plaques show an example of a zone of complete lysis by a given phage on a given bacterial strain.

b Number of lysed bacterial strains/total number of bacterial strains.

The *Lactobacillus* strains used met all the criteria for probiotics, i.e., tolerance to low pH and bile, susceptibility to antibiotics, and lack of resistance genes. They were also able to survive at 4 °C in an aerobic atmosphere for 10 days.

It should also be noted that the level of lipopolysaccharides (LPS) in the suppositories, determined in the Limulus amebocyte lysate assay, was between 10 and 25 EU/mL.

The results of the in vivo experiment showed a significant (*p* ≤ 0.05) therapeutic and preventive effect of the experimental procedure in calves, manifested as a reduction in diarrhea and rectal temperatures as well as an increase in the body weight of calves treated with the experimental medicine (Table 2).

**Table 2:** Comparison of the Therapeutic Effect of Suppositories in Calves

parameter	group 1 *n* = 6	group 2 *n* = 6	group 3 *n* = 6	group 4 *n* = 6
Average weight (kg)				
day 1	44.8 ± 5	43 ± 10	41.4 ± 8	42.4 ± 10
day 11	53.5 ± 6^a^	47.2 ± 6	53.2 ± 7^a^	49.2 ± 6
Average rectal temp. °C				
day 1	38.5 ± 0.6^a^	40.1 ± 0.3^c^	38.5 ± 0.3	40.5 ± 0.1^c^
day 2	38.5 ± 0.3	40.5 ± 0.2^c^	38.5 ± 0.2	39.8 ± 0.2
day 3	38.4 ± 0.3	40.2 ± 0.3^c^	38.3 ± 0.1	39.6 ± 0.1
day 4	38.4 ± 0.3	39.9 ± 0.2	38.6 ± 0.1	38.8 ± 0.2
day 5	38.5 ± 0.1^a^	39.8 ± 0.3^b^	39.2 ± 0.2^a^^,^^b^	38.7 ± 0.1
day 7	38.8 ± 0.1	39.8 ± 0.1^b^	38.8 ± 0.1^a^	38.8 ± 0.2^a^
day 11	38.9 ± 0.1	39.7 ± 0.2^b^	38.5 ± 0.1^a^	38.9 ± 0.1^a^
% of calves with diarrhea on last day of application of suppositories	0	20	0	0
mortality %	0	0	0	0
reduction in pathogenic *E. coli* log CFU/g	nd	no reduction	nd	0.3

a Significant difference (*p* ≤ 0.05) in comparison to Group 2.

b Significant difference (*p* ≤ 0.05) in comparison to first day of experiment.

c Significant differences (*p* ≤ 0.05) in comparison to other days of experiment. nd, not detected because no pathogenic strains of *E. coli* were observed in this group of healthy calves; Group 1: control healthy—healthy calves that did not receive medicine; Group 2: control positive—calves with diarrhea that did not receive medicine; Group 3: healthy calves that received medicine; Group 4: calves with diarrhea that received medicine.

The experimental procedure involving five-day administration of suppositories with three *E. coli* phages combined with *Lactobacillus* spp. in the treatment of neonatal calf diarrhea, with two applications on the first day of treatment, had an antibacterial effect. For the purposes of the treatment it was necessary to find bacteriophages with specific properties: resistance to pH < 4, a constant lytic titer, and a wide spectrum of activity. It is also significant that the experimental treatment was prepared with *E. coli* strains isolated from various housing systems, which means that it was not intended as a targeted therapy limited to phages isolated from a specific farm environment.

The results showed a beneficial effect of bacteriophages with probiotics, i.e., a reduction in clinical signs of diarrhea in calves, including the frequency of defecation and the absence of watery diarrhea. A positive effect was also manifested as a decrease in rectal temperature (<40 °C) on the second day after application of suppositories in calves with diarrhea. No shock or toxic reaction was observed in treated calves as a response to the LPS contained in the medicine or released by *E. coli*. This confirms observations made in other studies of the safety of bacteriophage therapy in humans and animals,^20,21^ whose authors reported that application of phage T4 did not affect the production of inflammatory cytokines or reactive oxygen species (ROS) by cells exposed to endotoxin. This provides new evidence of possible interactions between phages and mammalian cells, which is important for medical and veterinary therapy. The use of STX-producing strains as hosts for bacteriophages is of epidemiological significance, because they also pose a threat to humans as consumers of products derived from cattle. Moreover, they are a reservoir of antibiotic resistance genes.

The results obtained for the suppositories were favorable in terms of the stability of the lytic titers over 4 weeks of use and control of only pathogenic strains of *E. coli*. The bacteriophages also showed no antibacterial activity against commensal *E. coli* strains isolated from the calves in in vitro conditions.

The protective effect in calves was confirmed by the absence of signs of diarrhea during the next 4 weeks of rearing. The phage-probiotic therapy significantly reduced pathogenic *E. coli* strains isolated from calf feces 48 h after the first application. As a positive effect of the treatment, the bacteria were completely eliminated and no recolonization was observed for another 4 weeks, until the observations of the animals were completed.

In Group 4, the clinical signs were severe at the start of the experiment, but after application of suppositories the percentage of calves with diarrhea decreased to 20% during the first 24 h after first application of medicine. Bacteriophages were excreted by the calves in their feces for 2 weeks after the end of treatment, and their lytic titer was stable, ranging from 2.5 × 10^7^ PFU/mL to 1.9 × 10^8^ PFU/mL. It should also be emphasized that the phage therapy reduced the number of pathogenic *E. coli* strains by 0.3 log_10_ CFU/g of feces in calves with clinical diarrhea. The effectiveness of phage therapy in *E. coli* infections in newborn calves is determined by many factors, including the experimental infection scheme, the form of phage application, and the composition of the dose of bacteriophages. Johnson et al.^22^ and Sheng et al.^23^ have demonstrated that rectal administration of two phages, SH1 and KH1 (8.1 × 10^10^ PFU/mL), reduces the number of *E. coli* O157:H7 from steers by about 2 log CFU/mL. The authors also administered phages to calves and sheep via drinking water, at final daily concentrations of 1.8 × 10^6^ to 5.4 × 10^6^ PFU/mL, starting on day 0.

The route and form of bacteriophage application is a significant problem in obtaining effective antibacterial effects. For example, in a study by Rozema et al.,^24^ after rectal application of four doses of phages (10^10^ PFU/mL) the antibacterial effect against *E. coli* O157:H7 was lower than after oral administration. In the case of oral application of a bacteriophage cocktail with CEV1 and CEV2 to adult cattle and sheep, a significant reduction in diarrhea was observed within the first 24–48 h after application. This was accompanied by a 99% reduction in *E. coli* colonization in the rectum.^25^ In a study by Smith et al.,^26^ administration of a mixture of five phages with low in vitro virulence in the amount of 10^5^ PFU/mL by spraying the litter 10 min before challenge with *E. coli* B85 was no more successful than administration to infected calves. Stanford et al.^27^ reported a beneficial effect of five applications of a phage cocktail with probiotic strains in protective polymer capsules, with the highest antibacterial effect observed after 10-day application of boluses containing three phages with feed (1.13–1.81 × 10^9^ PFU/g). The authors also observed a preventive effect manifested as higher efficacy in the elimination of *E. coli* diarrheal infections.

**Figure 3:**
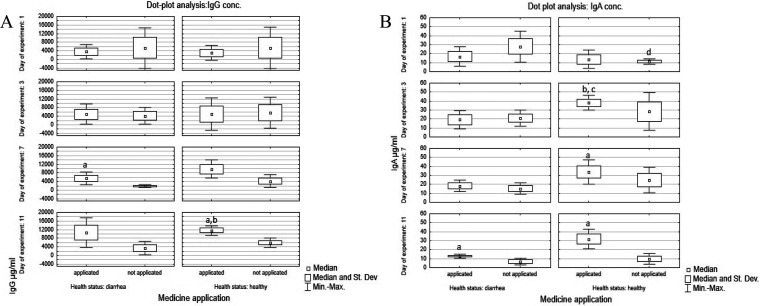
Dot plot analysis of average serum IgG and IgA concentrations in all experimental groups of calves. Legend A: a, significant difference at *p* ≤ 0.05 in comparison to untreated calves *p* = 0.04. Legend B: a, significant difference in comparison to untreated calves [*p* = 0.04]; post hoc effect analysis of variance, Levene’s test, significant differences at *p* = 0.029 in comparison to untreated calves; b, significant differences between heathy treated and diarrheic calves *p* = 0.048; c, significant differences between healthy treated and diarrheic treated calves *p* = 0.008; d, significant differences at *p* ≤ 0.05 between healthy untreated and diarrheic untreated calves *p* = 0.008

The medicine used in our study also had an immunomodulatory effect, involving a significant (*p* ≤ 0.05) increase in the humoral specific immune response, manifested as higher IgA and IgG concentrations, as well as nonspecific parameters, including IFNγ and lysozyme levels, in both diarrheic and healthy calves treated with suppositories. This may indicate an additive effect of phages and probiotics. The highest significant IgG level (16869.7 μg/mL) was observed in Group 3 on the last day of the experiment. A high, significant IgG concentration was also found on the seventh day of the experiment in calves with diarrhea that received suppositories (Group 4; Figure 3).

**Figure 4:**
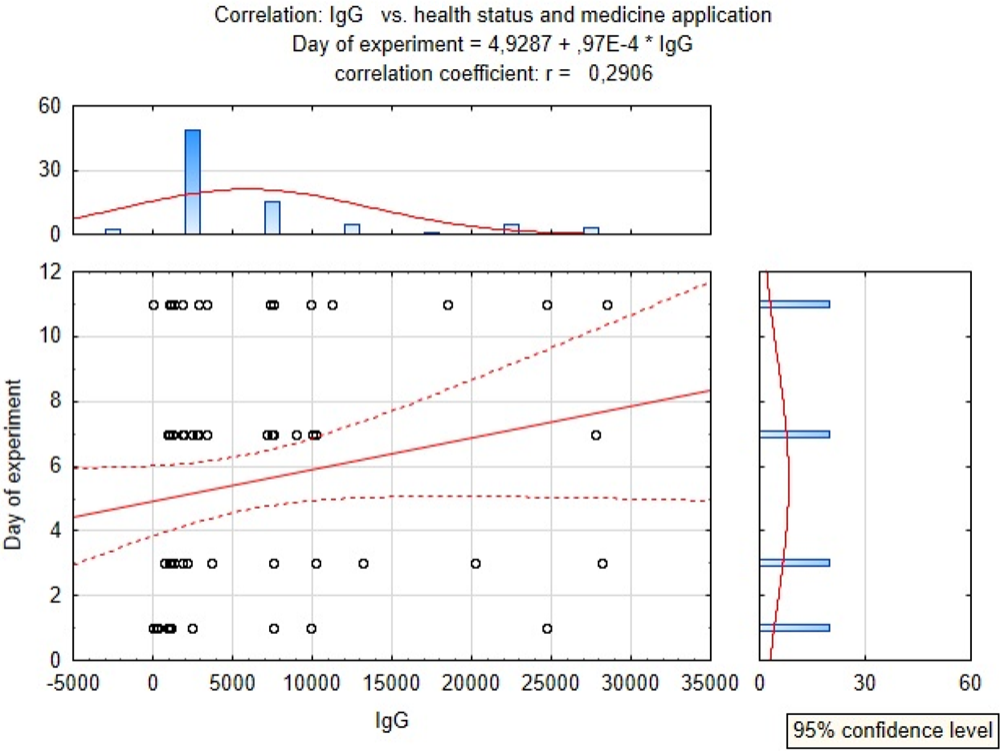
Correlation between IgG concentration, health status, and administration of medicine.

The correlation between the IgG level in the calves and medicine application was fairly low *r* = 0.29 (Figure 4).

**Figure 5:**
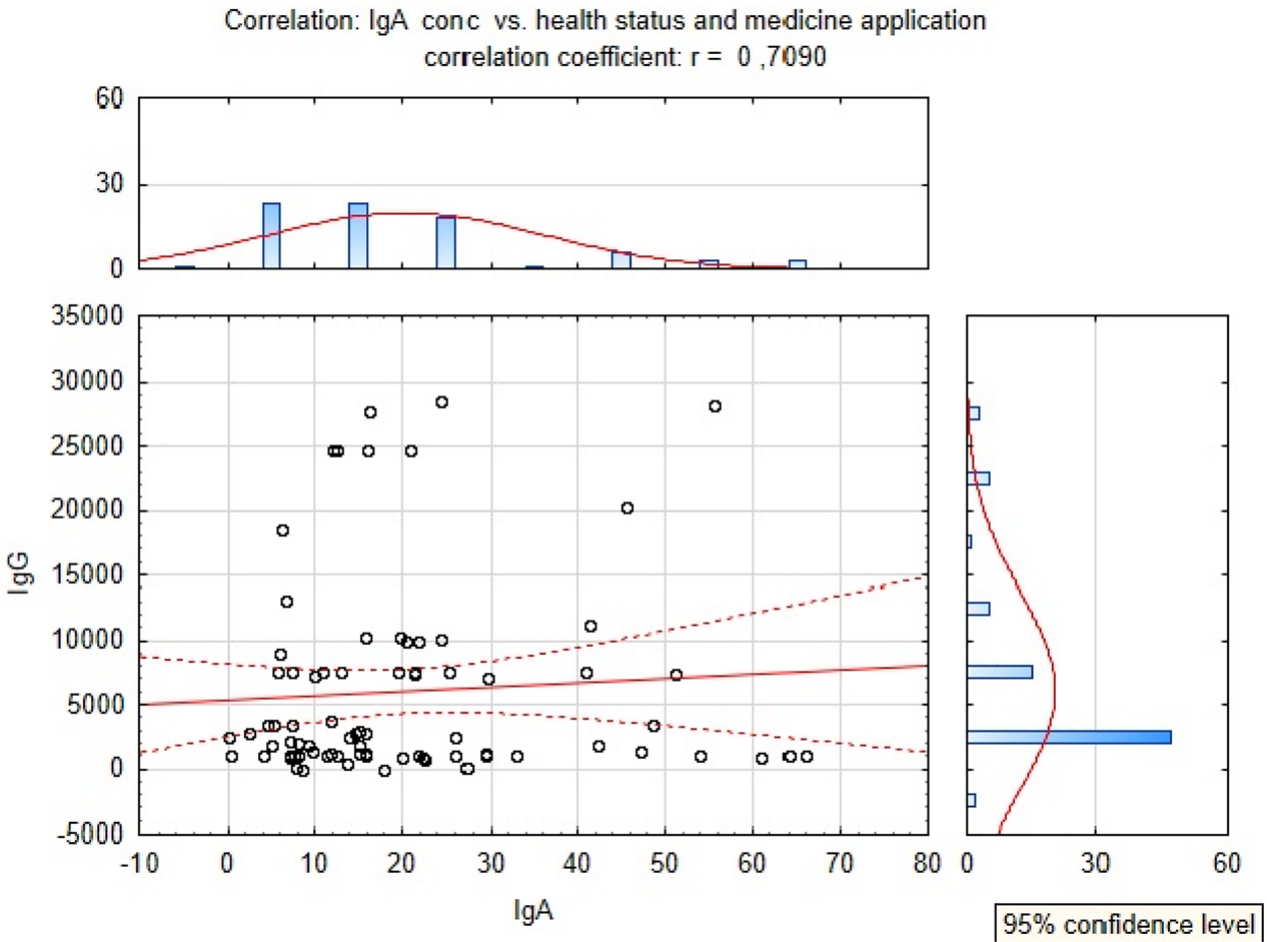
Correlation between IgA concentration, health status, and administration of medicine

There was also a significant (*p* ≤ 0.05) increase in the IgA level in the group of calves receiving medicine in comparison to the untreated animals, and these results were more significant than the results obtained for the IgG concentration. The highest concentration of IgA was observed in the group of healthy calves that received medicine on the third day, and this significantly (*p* ≤ 0.05) higher level in comparison to untreated calves was observed up to the last day of the experiment (Figure 5). In the case of calves with diarrhea receiving suppositories (Group 4), a significant increase (*p* ≤ 0.05) in the IgA concentration in comparison to untreated calves was observed on the last (11th) day of the experiment. The post hoc effect was significant (*p* ≤ 0.05) between the groups of calves which received medicine in comparison to the untreated animals.

A strong correlation was also observed between the IgA level and medicine application (*r* = 0.7) (Figure 5).

The average IgM concentration was very low in all experimental groups of calves, and the results were not statistically significant. The results obtained for IgM concentration were similar (>9 μg/mL) in all experimental groups of calves. Only in Group 4 was the highest mean concentration 15.54 μg/mL, but this result was not statistically significant.

Analysis of selected nonspecific immunological parameters, i.e., lysozyme and IFNγ levels, showed significant changes in calves which received medicine in comparison to calves from the control groups (Groups 1 and 2). It should be noted that IFN-γ participates in activation of lymphocytes in the antiviral response, which is associated with stimulation of macrophages to kill intracellular organisms (viruses, parasites, and mycoplasmas).^28^

**Figure 6:**
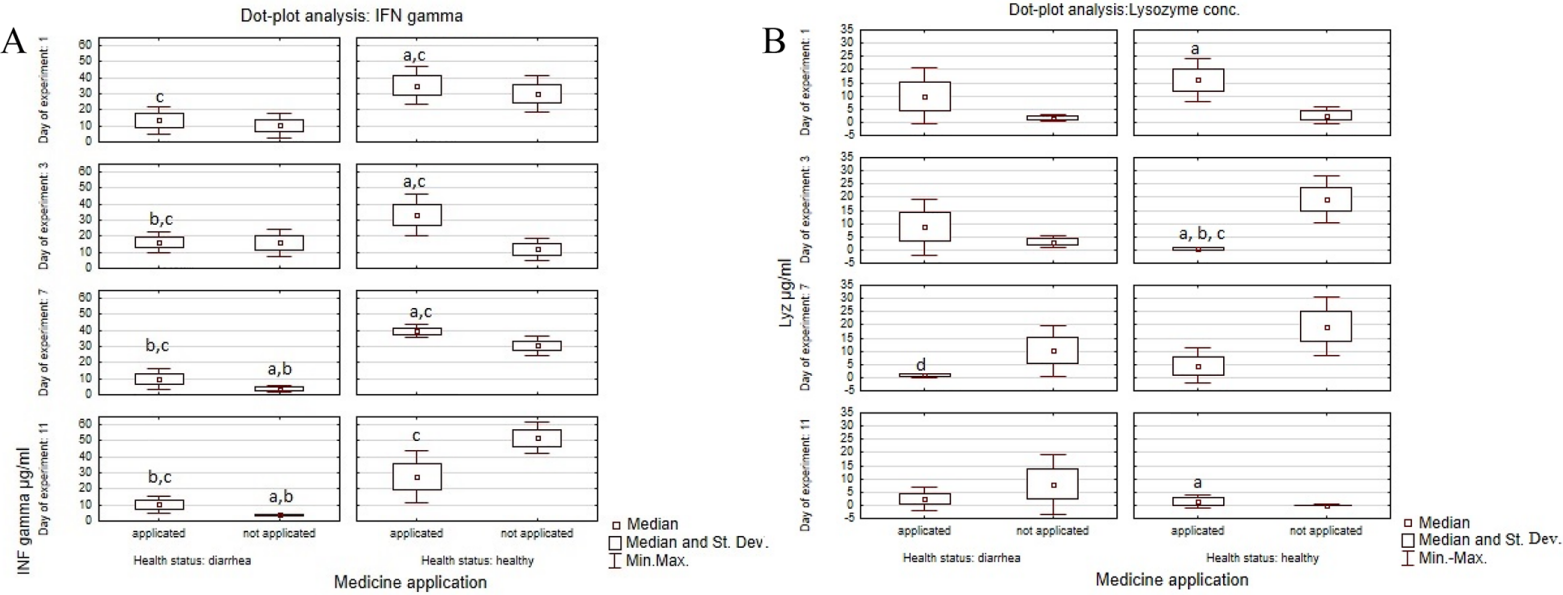
Dot plot analysis of average serum concentrations of lysozyme and INFγ in experimental groups of calves. Legend A: a, significant difference between healthy treated and healthy untreated calves *p* = 0.00001; b, significant differences between healthy untreated and diarrheic untreated calves *p* = 0.0001; c, significant differences between healthy treated vs diarrheic treated calves [*p* = 0.006]; d, significant differences between diarrheic treated calves vs untreated calves [*p* = 0.008]. Legend B: a, significant differences at *p* ≤ 0.05 between treated and untreated calves (*p* = 0.0018); b, significant differences at *p* ≤ 0.05 between healthy calves and calves with diarrhea (*p* = 0.008); c, significant differences at *p* ≤ 0.05 between treated healthy and treated diarrheic calves.

The results obtained for the serum lysozyme level showed a significant (*p* ≤ 0.05) increase (20.83 μg/mL) in the healthy treated calves. The results were statistically significant at *p* ≤ 0.05 in comparison to the healthy control calves during the first 7 days of the experiment. Significant differences at *p* ≤ 0.05 in the lysozyme level were also observed between calves with diarrhea treated with medicine (Group 4) and the control (untreated) calves with diarrhea (Group 2). Significant differences at *p* ≤ 0.05 in lysozyme concentration were also observed between Group 3 (healthy treated) and Group 1 (healthy untreated) and between the first group (healthy untreated) and the second group (diarrheic, untreated) (Figure 6).

The concentration of IFNγ was highest on all days of the experiment in the healthy calves that received suppositories (Group 3). A high concentration was also observed during the last 5 days of the experiment in the healthy untreated calves (Group 1, control). The results were significant at *p* ≤ 0.05 in comparison with the groups of calves with diarrhea, treated and untreated. The lowest IFNγ concentration (3.67 μg/mL) was found in calves with diarrhea in the positive control group (Group 2). These results were significant at *p* ≤ 0.05 in comparison to all experimental groups of calves on the last 5 days of the experiment. A significantly higher (*p* ≤ 0.05) IFNγ level was also observed in the healthy calves (Group 3) which received medicine in comparison to Group 4, calves with diarrhea treated with medicine (Figure 6). A low negative correlation was shown between the IFNγ level and the health status of calves in relation to medicine application (*r* = −0.02).

Górski et al.^29^ suggest that bacteriophages have the ability to translocate through the gastrointestinal mucosa to distant tissues and interact with immune cells. This could be crucial for their use in prophylaxis of diarrhea induced by bacteria and some viruses. Besides stimulation of the antiviral immune response, the bacteriophage treatment used in our study also had a significant protective effect on resistance of calves to diarrheal infections caused by pathogenic *E. coli*, which was confirmed by the absence of diarrhea during the next 3 weeks.

The prophylactic effect of phage-probiotic suppositories in the present study also translated into significant changes in Hp and SAA levels in treated calves. The acute phase response in calves based on SAA and Hp concentrations showed a significantly higher SAA level (236.062 μg/mL) in the control group in comparison to the calves with diarrhea (94.002 μg/mL). Similar APP changes have been observed by Pourjafar et al.,^30^ who apart from an increase in IFNγ and TNFα reported increased SAA and Hp levels in diarrheic calves.

**Figure 7:**
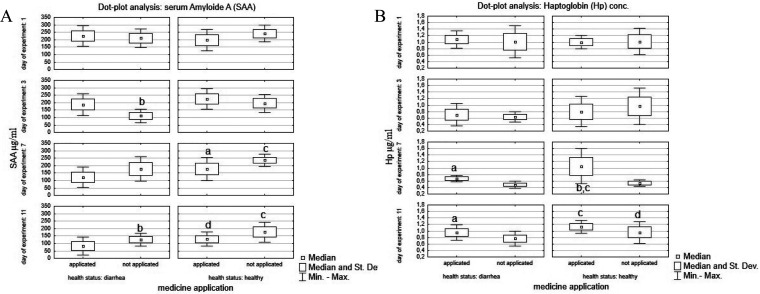
Dot plot analysis of average plasma SAA and haptoglobin concentration in experimental groups of calves. Legend A: a, significant differences at *p* ≤ 0.05 for healthy treated vs healthy untreated calves; b, significant differences for treated calves with diarrhea vs untreated calves with diarrhea; c, significant differences for healthy untreated calves vs diarrheic treated calves; d, significant differences at *p* ≤ 0.05 for healthy treated calves vs diarrheic treated calves. Legend B: a, significant differences in analysis of variance at *p* ≤ 0.05 for diarrheic treated vs diarrheic untreated calves *p* = 0.0054; b, significant differences for healthy treated vs healthy untreated calves *p* = 0.0013; c, significant differences for healthy treated vs diarrheic calves *p* = 0.005; d, significant differences at *p* ≤ 0.05 for healthy treated vs diarrheic untreated calves.

A significant difference at *p* ≤ 0.05 was also observed between healthy treated and untreated groups (Figure 7). The results obtained for Hp showed an increase in all experimental groups of calves. However, significant differences (*p* ≤ 0.05) in comparison to the control were observed only in calves with diarrhea. The correlation between APP levels in treated and untreated groups was at a moderate level of *r* = −0.43 (Figure 7).

In the present study, a significant increase in Hp and SAA levels was observed in healthy calves and calves with diarrhea that were not treated with phages. The results may indicate that the components of the suppositories together with LPS at the level of >20 EU/mL were not involved in the induction of an inflammatory reaction, which confirms the high safety level of the medicine. It is also worth noting that the concentrations of APP in all groups of calves were at detectable levels. According to Schroedl et al.,^31^ the values of these proteins in calves from directly after birth to even 10 days of age may indicate an internal response to stress factors. Seppa-Lassila et al.,^32^ however, suggest that serum Hp levels are nearly undetectable in healthy mature individuals, while concentrations up to 200 ng/mL are acceptable for healthy animals.

In the present study, the average Hp concentration in healthy and sick calves ranged from about 500 to 1600 ng/mL and clearly indicated the course of the inflammatory process resulting from diarrheal infection in sick calves and the effects of other environmental stressors. Despite such high Hp and SAA levels in the calves, in both cases a downward trend was observed in the concentrations of acute phase proteins, which is a positive indicator of the animals’ health status.

## Methods

The authors obtained approval for the experiment from the Local Animal Care Ethics Committee in Lublin (no. 37/2015).

Bacteriophages were obtained from 20 *Escherichia coli* isolates selected as hosts for bacteriophages showing anti-*E. coli* activity. Twelve Shiga-toxin-producing *E. coli* strains (No. 6, 10, 16, 21, 26, 27, 29, 39, 43, 22, 51, 55) isolated from calves were obtained from Prof. J. Osek of the National Veterinary Research Institute in Puławy, and eight *E. coli* isolates (No. C1, C2A, C28, IND, 771, 772, 4126, 527) were derived from calves with clinical signs of diarrhea. All necessary information about the strains used is contained in the work by Osek.^33^

Bacteriophages specific to Shiga-toxin *E. coli* were isolated from bovine feces. A total of 11 bacteriophages were chosen for detailed characterization: φ26, φ29, φ21, φ27, φ6, φ44, φ16, φ39, φ55, and φ51. This was the first step in selecting the bacteriophages expected to have the best properties for use in suppositories.

Three probiotic *Lactobacillus* spp. strains, obtained from our own collection of isolates from cattle, were used, including isolates from colostrum and from feces: *L. fermentum* (2 strains) and *L. salivarius* (1 strain). The probiotic properties of the *Lactobacillus* strains were determined on the basis of detection of H_2_O_2_ production, measurement of bacterial hydrophobicity, tolerance for acidic pH, bile tolerance, and bacterial survival in MRS in broth at 4 °C.^34,35^ The *Lactobacillus* strains used to develop the probiotic-phage preparation in the form of a suppository were deposited in the Polish Collection of Microorganisms, no. B/00169.

### Preparation of Bacteriophages

The phages were isolated according to Huff et al.^36^ The lytic properties and host ranges of the phages were determined by plaque assays on double-layer top agar plates. The control consisted of plates containing *E. coli* strains suspended in top agar. The plates were incubated overnight at 37 °C, and the results were scored as a clear zone of complete lysis (++), partial lysis with turbidity (+), or no lysis (−).^37^

The morphology of phages was determined with a transmission electron microscope (TEM) on negative-stained slides with 2% silicotungstate. The lytic properties and host ranges of the phages were determined by plaque assays on double-layer agar plates.^36^

Bacteriophage genome size was determined by pulsed field gel electrophoresis (PFGE).^38^ Chromosomes isolated from *Saccharomyces cerevisiae*, strain YPH80, supplied by Sigma, UK, were included in each gel and used as a PFGE marker. The wells were sealed with molten 1% (w/v) agarose and allowed to set, after which they were transferred to a Bio-Rad CHEF-DR II electrophoresis system (Bio-Rad, UK). Electrophoresis was performed in 0.5 × TBE at 6 V/cm for 18 h with incremental pulses of 2.2–54.2 s and with the buffer circulating at 14 °C. Gels were stained in 1 μg/mL of ethidium bromide, and images were captured with a ChemiDoc XRS Imager (Bio-Rad, UK) using Quantity One software.

The phage suspension was concentrated using PEG 8000. For this procedure, 30 mL of phage suspension was added to 8 mL of 20% PEG8000/2.5 M NaCl buffer and then mixed by vortexing and refrigerated. Finally, it was suspended in 1 mL of TM buffer and refrigerated at −80 °C.

### Treatment Protocol

The study was carried out on 24 female HF calves, aged from 2 to 7 days and weighing from 35 to 46 kg, with clinical signs of bacterial diarrhea (rectal temp >39.9 °C, depression, feces with changed color and liquid consistency). The experiment was conducted in late October and early November 2018. All calves were kept in individual pens on litter during the first 3 weeks after birth. In subsequent weeks, the calves were kept in groups of no more than 3 animals in conditions compliant with Council Directive 2008/119/EC. After the colostrum period all calves were fed with a milk replacer (Polmass.eu, PL) according to the manufacturer’s instructions. The calves were randomly divided into four groups of six calves each: Group 1, healthy control, calves that received no medicine; Group 2, positive control, calves with diarrhea not treated with medicine; Group 3, healthy calves that received medicine (this group was necessary for evaluation of the prophylactic effect of the suppositories); Group 4, calves with diarrhea that received medicine. The animals received suppositories with *Lactobacillus* spp. and bacteriophages specific for pathogenic *E. coli* strains *per rectum* for 5 days. On the first day, the calves received the suppositories twice—in the morning and 12 h later; subsequently they were administered only once a day. The health status of the calves (body temperature, fecal consistency, and mood change, e.g., depression) was observed for 11 days after the first day of application of suppositories. Clinical status was scored on a scale of 0 (normal) to 3 (severe) symptoms according to Romanowski et al.^39^

Total enumeration of pathogenic *E. coli* strains was carried out using the horizontal method for the enumeration of beta-glucuronidase-positive *E. coli* according to ISO 16649–3.^40^

The suppositories were prepared at the Department of Pharmacology and the Sub-Department of Veterinary Prevention and Avian Diseases, University of Life Sciences in Lublin (Patent no P.424314). The medicine contains a phage cocktail with log 10^9^ PFU/mL of three bacteriophages specific for pathogenic *E. coli* strains (φ26, φ27, and φ29) and three probiotic *Lactobacillus* spp. strains—4a *L. salivarius*, 6b *L. fermentum*, and 66a *L. fermentum*—at optical density OD = 7.0 (∼5 × 10^9^ CFU/mL).^41^ Rectal suppositories were used because many studies have found oral application of phages to be unsuccessful due to gastric acid conditions.

The strongly lytic phages obtained were tested to confirm their wide spectrum of lytic activity against Stx and K99 *E. coli* strains. To confirm the lack of antibacterial activity against commensal strains, the phages used in the medicine were also tested on 150 commensal *E. coli* strains from our institute collection.

To test the activity of the biological agents included in the suppositories, they were tested according to the following protocols:

- Melted suppositories were spread on an LB agar plate cultured with *E. coli* strains no. 26, 27, and 29 to test the activity of bacteriophages; the plates were incubated for 24 h at 37 °C.
- One suppository was melted in 2 mL of MRS broth warmed to 40 °C; the total volume was 3.5 mL. The solution was serially diluted in MRS broth, and 500 μL of each dilution was spread onto MRS agar; plates were incubated at 37 °C, 5% CO_2_. After 48 h the viability of the bacteria was evaluated based on the number of colonies.

### Specific and Nonspecific Immune Responses in Calves

To evaluate the specific and nonspecific immune response of calves, the concentrations of immunoglobulins (IgA, IgM, and IgG), lysozyme, and IFNγ in the calf sera were determined using ELISA assays. Selected acute phase proteins (APP), i.e., serum amyloid A and haptoglobin, were also tested using ELISA to evaluate the calves’ response to inflammation.

For this purpose, blood from calves was collected into EDTA-free tubes for sera and EDTA tubes on days 1, 3, 7, and 11 of the experiment. Sera and plasma samples were kept at −20 °C until analysis. The tests were carried out using commercial ELISA kits according to the manufacturers’ instructions: Cusabio (China) for immunoglobulin concentration; Tridelta (Ireland) for SAA and haptoglobin concentration; BioSource (Sweden) for lysozyme; and Mabtech AB (Sweden) for IFNγ.

To identify the presence of pathogenic *E. coli* strains in calves and the duration of bacteriophage excretion from the calves, fecal samples were collected on the same days as the blood samples.

The results were statistically analyzed using Statistica 10.0. One-way analysis of variance (ANOVA) was used to compare differences between groups. The post hoc effect was determined using the Tukey test. Correlation analysis of selected parameters was performed using the Pearson correlation coefficient. Significance of differences was reported for *P* ≤ 0.05.

## Conclusions

To sum up, the experimental phage therapy is useful in the control and prevention of bacterial infection in young calves. The most important effect was stimulation of a specific and nonspecific humoral immune response. This was the first treatment attempted and may be the first step in further research on the control of diarrhea induced by *E. coli* in calves. Due to the lack of a species barrier, the procedure can potentially be used in other animal species and in humans.

## Abbreviations

APP: acute phase proteins
CFU: colony-forming unit
EDTA: ethylenediaminetetraacetic acid
ELISA: enzyme-linked immunosorbent assay
EPEC: enteropathogenic *E. coli* strains
ETEC: enterotoxigenic *E. coli* strains
HF: Holstein–Friesian
Hp: haptoglobin
LPS: lipopolysaccharide
MRS: de Man, Rogosa, and Sharpe
NaCl: natrium chloride
PCR: polymerase chain reaction
PFGE: pulsed field gel electrophoresis
PFU: plaque-forming unit
ROS: reactive oxygen species
SAA: serum amyloid A
Stx: Shiga toxin
TE buffer: Tris EDTA buffer
TM buffer: Tris Magnesium buffer.
